# Cultured mycelium *Cordyceps sinensis* protects liver sinusoidal endothelial cells in acute liver injured mice

**DOI:** 10.1007/s11033-014-3031-y

**Published:** 2014-01-19

**Authors:** Yuan Peng, Qian Chen, Tao Yang, Yanyan Tao, Xiong Lu, Chenghai Liu

**Affiliations:** 1 Institute of Liver Diseases, Shuguang Hospital Affiliated to Shanghai University of Traditional Chinese Medicine, 528 Zhangheng Road, Pudong New Area, Shanghai, 201203 China; 2 Central Laboratory, Shanghai Xuhui Central Hospital, Shanghai, 200031 China; 3 Shanghai Key Laboratory of Traditional Chinese Clinical Medicine, Shanghai, 201203 China; 4 Shanghai University of Traditional Chinese Medicine, Shanghai, 201203 China; 5 E-Institute of TCM Internal Medicine, Shanghai Municipal Education Commission, Shanghai, 201203 China

**Keywords:** Mycelium *Cordyceps sinensis*, Liver sinusoidal endothelial cells (LSECs), Liver injury, Hepatic sinusoid, MMP-2/9, Oxidative stress

## Abstract

Cultured mycelium *Cordyceps sinensis* (CMCS) was widely used for a variety of diseases including liver injury, the current study aims to investigate the protective effects of CMCS on liver sinusoidal endothelial cells (LSECs) in acute injury liver and related action mechanisms. The mice were injected intraperitoneally with lipopolysaccharide (LPS) and d-galactosamine (D-GalN). 39 male BABL/c mice were randomly divided into four groups: normal control, model control, CMCS treatment and 1,10-phenanthroline treatment groups. The Serum liver function parameters including alanine aminotransferase (ALT) and aspartate aminotransferase (AST) levels were assayed with the commercial kit. The inflammation and scaffold structure in liver were stained with hematoxylin and eosin and silver staining respectively. The LSECs and sub-endothelial basement membrane were observed with the scanning and transmission electronic microscope. The protein expressions of intercellular adhesion molecule-1 (ICAM-1) and vascular cell adhesion molecule-1 (VCAM-1) in liver were analyzed with Western blotting. Expression of von Willebrand factor (vWF) was investigated with immunofluorescence staining. The lipid peroxidation indicators including antisuperoxideanion (ASAFR), hydroxyl free radical (·OH), superoxide dismutase (SOD), malondialdehyde and glutathione *S*-transferase (GST) were determined with kits, and matrix metalloproteinase-2 and 9 (MMP-2/9) activities in liver were analyzed with gelatin zymography and in situ fluorescent zymography respectively. The model mice had much higher serum levels of ALT and AST than the normal mice. Compared to that in the normal control, more severe liver inflammation and hepatocyte apoptosis, worse hepatic lipid peroxidation demonstrated by the increased ASAFR, ·OH and MDA, but decreased SOD and GST, increased MMP-2/9 activities and VCAM-1, ICAM-1 and vWF expressions, which revealed obvious LSEC injury and scaffold structure broken, were shown in the model control. Compared with the model group, CMCS and 1,10-phenanthroline significantly improved serum ALT/AST, attenuated hepatic inflammation and improved peroxidative injury in liver, decreased MMP-2/9 activities in liver tissue, improved integration of scaffold structure, and decreased protein expression of VCAM-1 and ICAM-1. CMCS could protect LSECs from injury and maintain the microvasculature integration in acute injured liver of mice induced by LPS/D-GalN. Its action mechanism was associated with the down-regulation of MMP-2/9 activities and inhibition of peroxidation in injured liver.

## Introduction

The liver contains a myriad of network of vessels, including microvascular system, nourishing the parenchymal cells. The hepatic sinusoid is unique, dynamic microvascular structure that serves as the principal site of exchange between the blood and the perisinusoidal space. Liver sinusoidal endothelial cells (LSECs) are the major cell types in hepatic sinusoid, with the characteristic of fenestration in cell and endothelial matrix, the latter is called as “endothelial basement membrane”, which in normal liver consists of non-fibril-forming collagen including type IV collagen and proteoglycan and glycoproteins. This normal endothelial matrix is critical for maintaining the differentiated functions of resident liver cells such as hepatocytes, hepatic stellate cells and LSECs, etc. LSECs injury occurred early when the liver suffered the insults, since it was exposed to higher blood levels than systematic concentrations toxins, while many toxins or metabolites absorbed from gastrointestinal tract were taken up into portal venous blood. In addition, the detachment of LSECs from endothelial matrix plate was critical component of injuries, while the inflammatory cells could improve matrix metalloproteinases-2/9 (MMP-2/9) secretion at earlier liver injury and led to LSECs detachment.

Recently, traditional Chinese medicine (TCM) is becoming an increasingly popular form of complementary medicine in western countries. *Cordyceps sinensis* (Berk.) Sacc., as a well-known tonic herb in TCM, is a highly valued fungus in China as playing an important role in regulating the disorder of biological activity. In recent years, cultured mycelium *Cordyceps sinensis* (CMCS) was successfully developed and widely used as the alternative of natural *Cordyceps sinensis* (Berk.) Sacc [1]. Accumulated evidences from both animal and human studies indicated that *Cordyceps sinensis* was capable of anti-fibrosis [2] and anti-inflammation [3]. Furthermore, it acted as an anti-tumor, anti-proliferative, anti-metastatic, insecticidal and anti-bacterial compound [4]. In our previous studies, CMCS showed superior effects of anti-liver injury [5]. Simultaneously, it was able to decreasing the injury and phenotype shift of LSECs [6].

In the study, we observed the effect of CMCS on hepatic sinusoid in liver injury, including LSECs phenotype, basement membrane and matrix metalloproteinases-2/9 (MMP-2/9) activities, in order to investigate CMCS action mechanism against liver injury.

## Materials and methods

### Reagents

Lipopolysaccharide (LPS), d-galactosamine (D-GalN) and 1,10-phenanthroline were obtained from Sigma Chemical Co. All other chemicals and solvents were of the highest grade commercially available.

### Drug preparation

**Fig. 1:**
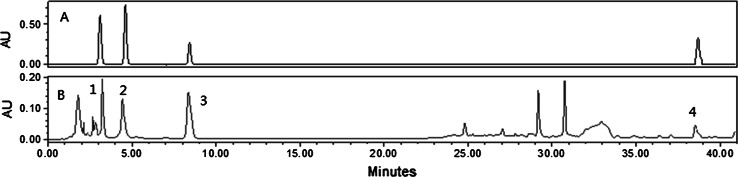
Chromatogram of mixed standard (*a*) and CMCS (*b*). The peak No. refer to standard content, *1* vernine (1.15 ± 0.01 %), *2* uridine (1.24 ± 0.02 %), *3* adenosine (0.14 ± 0.00 %), *4* ergosterol (1.04 ± 0.01 %)

The powder of CMCS (1.0 kg), were provided from Shanghai Sundise Chinese Medicine Technology Development Co., Ltd, and were extracted successively three times with 6 l of water under reflux, and the combined extract were concentrated under vacuum at 50 °C and then dried by lyophilization to afford the extraction of CMCS (150 g). The end extraction contained 150 mg/ml of CMCS drug. The quantitative analyses of active compounds were determined by high performance liquid chromatography (HPLC), which performed on Waters2695 systerm (Waters Corporation, Milford, USA), equipped with an Waters PDA2996 analyzer. Alliance software was used for the data analysis and the result was shown in Fig. 1.

### Animals

Male BABL/c mice weighing 18-22 g, specific-pathogen-free (SPF) level, were provided by Sino-British SIPPR/BK Lab Animals (Shanghai, China). They were housed in a room under controlled temperature (22–25 °C), humidity (46-52 %), and lighting (12-hour artificial light and dark cycle), with free access to tap water and mouse chow. The standard diet pellets contained not less than 20 % protein, 5 % fibers, 3.5 % fats, and 6.5 % ash and vitamins mixture. This experiment was conducted according to the local ethical guidelines (Shanghai University of TCM, Shanghai, China).

### Experimental design

All the mice were randomly divided into four experimental groups: normal control (*n* = 8), model control (*n* = 12), CMCS treatment (*n* = 10) and 1,10-phenanthroline treatment group (*n* = 9). Mice in normal control and model control were administered orally by gavage with distilled water once a day, for four consecutive days at a dose of 4 ml/kg of body weight, respectively. Mice in CMCS treatment group were treated orally with CMCS at a daily dose of 120 mg/kg of body weight at the same time, which was equivalent to 10 times of the 60 kg adult dose. In 1,10-phenanthroline treatment group, mice were intraperitoneally injected with 1,10-phenanthroline twice a day, for four consecutive days at a daily dose of 25 ml/kg of body weight. 1,10-phenanthroline was used as the positive control medicine and its dosage was based on our previous trial experiment. On the fourth day, 1 h after the last intragastrical administration, the normal control group was received intraperitoneal injections of saline at a dose of 2 ml/kg of body weight. The other mice were received intraperitoneal injections of LPS and D-GalN at the dose of 10 μg/kg and 900 mg/kg of body weight respectively [7]. All mice were sacrificed 7 h after the last injection.

### Serum levels of liver function

Measurement of serum alanine aminotransferase (ALT) and aspartate aminotransferase (AST) activities were measured by using SpectraMax-M5 Multifunctional microplate reader (Molecular Devices, Inc., Sunnyvale, California, USA) according to the manufacture’s instructions. Liver function tests kits were supplied by Nanjing Jian Cheng Bioengineering Institute (Nanjing, China).

### Parameters for peroxidative damage in liver

Hepatic homogenates were centrifuged at 3,000 r/min for 20 min at 4 °C. Supernatants were immediately collected and assayed for enzyme activities. Levels of antisuperoxideanion (ASAFR), hydroxyl free radical (·OH), superoxide dismutase (SOD), malondialdehyde (MDA) and glutathione *S*-transferase (GST) were assayed according to the protocols of kits purchased from NanJing Jian Cheng Bioengineering Institute. All these parameters were expressed by gram protein which was determined by the BCA protein assay kit (Pierce, Thermo Scientific, Rockford, USA) according to the manufacturer’s protocol using bovine serum albumin as a standard.

### Histopathology

Liver specimens were fixed in 10 % formaldehyde solution and dehydrated in a graded alcohol series, embedded in paraffin blocks, and cut into 4 μm thick slices. Slices for histopathological examination were stained by using standard procedure of hematoxylin and eosin (H&E) and silver, respectively.

### TUNEL staining

Fresh liver tissue was fixed in 10 % formaldehyde solution, embedded in paraffin, and sliced into 4-μm sections. Induction of apoptosis was measured by terminal deoxynucleotidyl transferase-mediated deoxyuridine triphosphate nick-end labeling (TUNEL) (ApopTag Peroxidase *In Situ* Apoptosis Detection Kit, S7100, Merck Millipore, Chemicon International, Inc, Billerica, MA). The apoptotic cells were stained brown, and nuclei were counterstained with Hematoxylin. TUNEL staining were analyzed with light microscope (Olympus BX40, Japan). To quantify the histologic findings, semi-quantification data for apoptosis in TUNEL staining were determined with the computer-assisted image analysis system.

Besides, some of the liver tissues were embedded in OCT compound and stored at −70 °C. The frozen tissue block was sectioned using a crytome (Leica CM1850, Germany). Ten-micrometer-thick cryostat sections were cut and transferred to poly-l-lysine-coated slides. Cell apoptosis detection was performed with the one step TUNEL kit (Beyotime, Jiangsu, China) according to the manufacturer’s protocol. Briefly, the sections were permeabilized with 0.1 % Triton X-100 for 2 min at 4 °C followed by TUNEL for 1 h at 37°. After washing, tissues were double stained with 4′,6-diamidino-2-phenylindole (DAPI) to visualize the nuclei. Images were obtained using a confocal microscope (Fluoview FV10i, Olympus, Japan) equipped with the Ultraviolet/Visible light LD laser combination. Photographs were taken with Olympus confocal software.

### Liver perfusion and processing for ultrastructural analysis

Livers were thoroughly cleared by perfusion with phosphate-buffered saline (PBS) of room temperature through the portal vein at a flow rate of 3 ml/min. One minute later, 2.5 % glutaraldehyde was perfused for an additional one minute at the same flow rate [8]. Subsequently, livers were carefully removed and quickly immersed in 2.5 % glutaraldehyde for 48 h at 4 °C as described previously [9].

For transmission electron microscope (TEM), several 1-mm^3^ cubes were harvested from the liver, washed three times in PBS, and fixed in aqueous 1 % osmicacid, 1 % potassium hexacyanoferrate (III) Red prussiate of potash for 1 h. After another three times washes, blocks were dehydrated through a graded series of 30–100 % ethanol, 100 % propylene oxide, and infiltrated for 1 h in a 1:1 mixture of propylene oxide: Polybed 618 epoxy resin (Shanghai Resin Factory Co. Ltd., Shanghai, China). After several changes of 100 % resin over 24 h, blocks were embedded in molds and cured at 37 °C overnight, followed by additional hardening at 65 °C for 48 h. Ultrathin (60 nm) sections were collected onto 200-mesh copper grids, stained with 2 % uranyl acetate in 50 % methanol for 10 min and 1 % lead citrate for 7 min, respectively. Sections were photographed with a Tecniai-12 transmission electron microscope (Philips, Amsterdam, The Netherlands).

For scanning electron microscopy (SEM), the 3-mm-thick fragments were cut from the fixative, washed three times with PBS, and then immersed in aqueous 1 % Osmicacid for 2 h. After three times washes in PBS, slices were dehydrated through a graded series of 30–100 % ethanol. Before critical point drying, washing with absolute ethanol was necessary. Slices were mounted onto aluminum stubs, sputter coated with gold/palladium, and viewed in a XL-30 scanning electron microscope (Philips, Amsterdam, the Netherlands) at 20.0 kV [10].

### Immunofluorescence staining

Frozen tissues embedded in OCT compound were cut into ten-micrometer thick and fixed in 4 % formaldehyde (Dingguo, Shanghai, China). After washing, sections were incubated with primary antibody for 1 h at 37 °C in a moist chamber. Rabbit anti-human von Willebrand Factor antibody (ab6994) was purchased from Abcam and used as the primary antibody at a dilution of 1:200 to examine the integrity of endothelial cells. To visualize the primary antibodies, cells were stained with Cy3-conjugated secondary antibodies. After washing, cells were double stained with DAPI to visualize the nuclei. Images were obtained using a confocal microscope and photographs were taken with Olympus confocal software. The semi-quantification data for vWF protein level in the liver tissue were determined with the computer-assisted image analysis system.

### Liver MMP-2/9 activities assay

MMPs activities in liver tissue were analyzed by gelatin zymography and in situ fluorescent zymography respectively. MMPs zymography assay was modified as previously described [11]. Briefly, liver tissues were homogenized and supernatant was aliquoted according to protein concentrations determined as the same procedures mentioned above. Aliquots (30 μg protein/lane) of liver tissue were prepared by dilution in the zymogram sample buffer (5 ×) containing 0.4 mol/l Tris, pH 6.8, 5 % SDS, 20 % glycerol and 0.03 % bromphenol blue, separated with electrophoresis in 10 % SDS-PAGE containing 1 mg/mL gelatine as a substrate under non-reducing conditions. Afterwards, gels were washed in the reaction buffer containing 50 mM Tris–HCl, 5 mM CaCl_2_, 1 μM ZnCl_2_ and 2.5 % Triton-X 100 (pH 7.5). The reaction buffer was changed to a fresh one, and gels were incubated at 30 °C for 18 h. Gelatinolytic activity was visualized by staining the gels with 0.1 % Coomassie brilliant blue G-250, destained with 30 % methanol/20 % acetic acid water and destained with 30 % methanol/10 % acetic acid respectively. Clear zones in the background of blue staining exhibit the presence of gelatinase activities. The intensity of the bands was scanned and assayed by Furi Gel Image software (Furi, Shanghai, China).

Fluorescent in situ zymography of liver section was performed according to Ben Wielockx’s methods [12] with modifications. Briefly, 1 mg/ml fluorescein-conjugated gelatine (Molecular Probes, USA) solution is diluted 1: 10 in the agarose-containing solution. Liver sections were mounted to the glass slide with the gelatin agarose mixture and incubated with 40 μg/ml in 0.5 mol/l Tris–HCl (pH 7.6), 50 mmol/l CaCl_2_ and 1.5 mol/l NaCl for 6 h at 37 °C. Sections were washed three times with water, and subsequently Nuclei was counterstained by adding Hoechst (Beyotime, Haimen, China). Gelatinase activity in situ was visualized using Olympus fluorescent microscopy.

### Western blot analyses

Western blot analyses were performed essentially as described [13]. Snap-frozen liver tissues were homogenized in RIPA lysis buffer containing 150 mM NaCl, 1 % NP-40, 0.1 % SDS, 50 mM Tris–HCl pH 7.4, 1 mM EDTA, 1 mM PMSF and 1× complete mini (Roche). Lysates were centrifuged at 10,000 g at 4 °C for 15 min to separate cytosolic-enriched supernatant from nuclei- and membrane-enriched pellets. Protein concentrations were determined using BCA protein assay kit mentioned above. Equal amount of proteins were separated by 10 % SDS gel electrophoresis (SDS-PAGE) under denaturing and non-reducing condition, and transferred to nitrocellulose membrane. Even transfer was confirmed by staining with 0.2 % Ponceau Red S in 3 % trichloroacetic acid. Membranes were blocked with 5 % nonfat milk in TBST (20 mM Tris–HCl, pH 7.5, 150 mM NaCl, 0.1 % Tween 20) at room temperature for 1 h, incubated with primary antibody ICAM-1(1:1,000; Santa Cruz Biotechnology, CA) and VCAM-1 (1:1,000; Santa Cruz Biotechnology, CA) at 4 °C overnight, respectively. After washing with TBST, blots were incubated with horseradish-coupled secondary antibody in wash buffer. Signals were developed with the immunoreactive bands were visualized using ECL kit (Upstate Biotechnology, Lake Placid, NY) according to the manufacturer’s instructions and quantified using a ChemiDoc image analyzer (Bio-Rad, Hercules, CA).

### Statistical analysis

All data were expressed as mean ± standard deviation (SD). Differences between the groups were assessed by nonparametric One-way analysis of variance (ANOVA) followed by the least significant difference (LSD) post hoc tests. Statistical significance was taken at the *p* < 0.05 level.

## Results

### CMCS alleviated LPS/D-GalN induced hepatic inflammation and hepatocellular apoptosis.

**Fig. 2:**
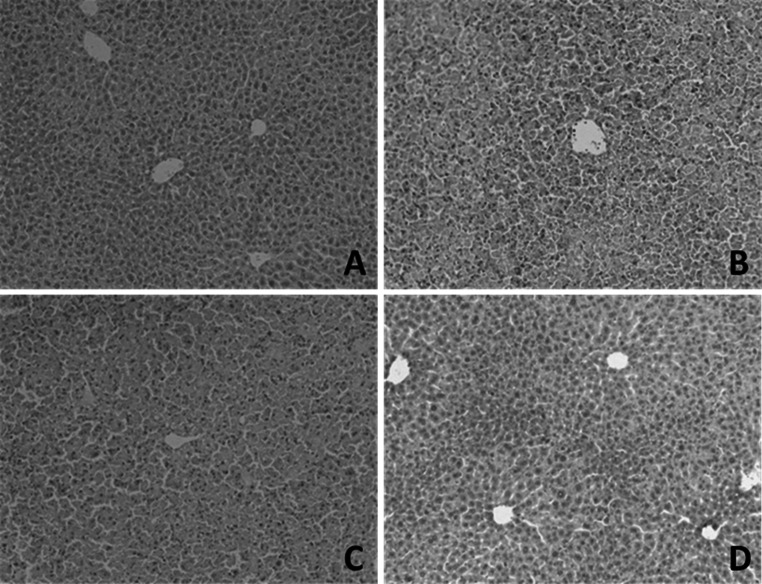
Effects of CMCS and 1,10-phenanthroline on liver inflammation in LPS/D-GalN-induced acute hepatic injury in mice. **a** Normal lobular architecture and orderliness arranged hepatocytes were observed in normal control group. **b** Obvious hepatic sinusoid bleeding and necrotic hepatocytes with widely mononuclear cell infiltration were seen in model control group. **c** Milder hepatic lesions and necrotic hepatocytes were observed in CMCS treatment group when compared with that in model group. **d** Intact hepatic structure, with obviously faint sporadic hepatic lesions, was noticed in 1,10-phenanthroline treatment group. H&E staining ×200

**Fig. 3:**
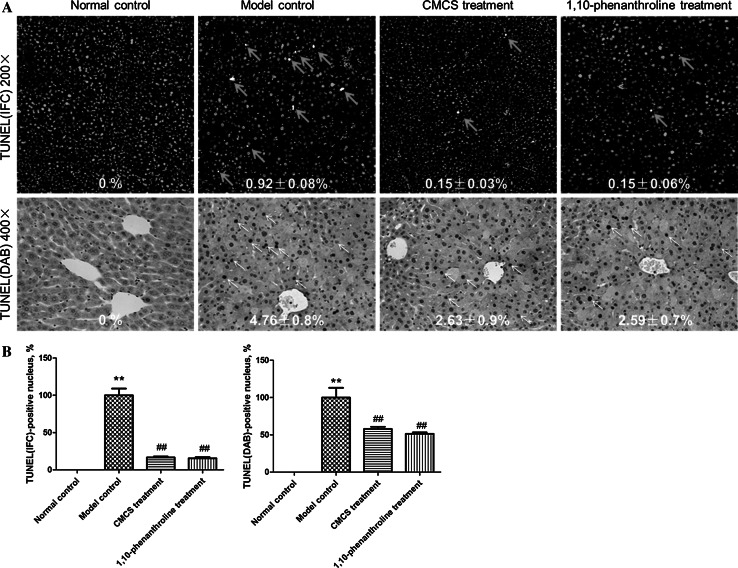
Effects of CMCS and 1,10-phenanthroline on apoptosis of hepatocytes in LPS/D-GalN-induced acute hepatic injury in mice. **A** In frozen liver tissue sections, TUNEL-positive cells (*red*) was scarcely observed in normal liver tissue, while apoptotic cells (*red*) marked with *orange arrows* were obviously observed in model control group. Fewer apoptotic cells were detected in CMCS and 1,10-phenanthroline treatment group respectively than that in the model control. In paraffin-embedded hepatic specimen, TUNEL-positive cells was scarcely observed in normal control, while apoptotic hepatocytes (*brown* or *deep brown*) marked with *red arrow* were obviously observed in model control. Apoptosis of hepatocytes in CMCS and 1,10-phenanthroline treatment group were decreased respectively in comparison with that in the model control. **B** Semi-quantification data for apoptosis in TUNEL staining were calculated in comparison with the model control (100 %) and were shown as proportion of TUNEL-positive nucleus for all nucleus. **p < 0.001, compared with normal control; ^##^ *p* < 0.001, compared with model control. (Color figure online)

In order to evaluate the effect of CMCS on LPS/D-GalN induced acute liver injury, serum levels of ALT and AST were determined. After LPS/D-GalN treatment, serum ALT and AST activities significantly elevated in mice (Table 1). In the vicinity of hepatic lesions, particularly alongside the lesion border, pronounced apoptotic bodies, necrotic and cytolytic hepatocytes with widely mononuclear cell infiltration were shown in the model mice (Figs. 2b, 3). Obviously, CMCS treatment group significantly decreased the elevated levels of ALT and AST activities (Table 1), ameliorated liver inflammation (Fig. 2c) and alleviated hepatocellular apoptosis in extent (Fig. 3) in comparison to the LPS/D-GalN treated group. Mice treated with 1,10-phenanthroline showed weak hepatic lesions alongside the central vein, with few apoptotic hepatocytes (Fig. 3).

**Table 1:** Effects of CMCS and 1,10-phenanthroline on serum liver function parameters in LPS/D-GalN-induced acute hepatic injury in mice ($\overline{x} \pm {\text{s}}$)

Group	*n*	ALT (IU/l)	AST (IU/l)
Normal control	8	11.52 ± 6.62	28.81 ± 16.33
Model control	12	145.7 ± 2.09*	148.17 ± 7.83*
CMCS treatment	10	77.97 ± 29.95^##^	91.30 ± 20.51^#^
1,10-Phenanthroline treatment	9	55.37 ± 31.85^##^	63.79 ± 28.03^##^

* *p* < 0.05, compared with normal control; ^#^ *p* < 0.05, ^##^ *p* < 0.001, compared with model control

### CMCS attenuated LPS/D-GalN induced hepatic scaffold structure broken

**Fig. 4:**
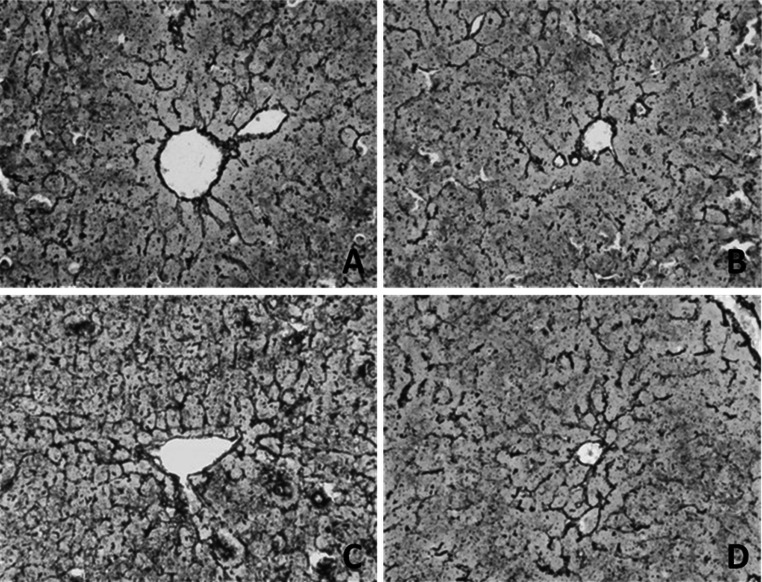
Effects of CMCS and 1,10-phenanthroline on scaffold structure in LPS/D-GalN-induced acute hepatic injury in mice. **a** Hepatic scaffold structures in normal mice were intact. **b** Hepatic scaffold structures in LPS/D-GalN-induced acute hepatic injury in mice were broken, with fragmentized silver nitrate deposits scattering in hepatic tissue. **c** Broken hepatic scaffold structure obviously decreased in liver sections in CMCS treatment mice. **d** Hepatic scaffold structure were scarcely broken in 1,10-phenanthroline treatment group. Silver staining ×400

Liver tissues in normal control showed intact scaffold structure with black continuous reticular fibers paralleled the hepatic cords (Fig. 4a). Obviously, liver sections of model mice exhibited severe broken hepatic scaffold structure (Fig. 4b) with fragmentized silver nitrate deposits scattering in hepatic tissue. By contrast, CMCS treatment and 1,10-phenanthroline treatment maintained hepatic scaffold structure in extent, respectively (Fig. 4c, d).

### CMCS protected LPS/D-GalN induced liver sinusoid endothelial cell injury

**Fig. 5:**
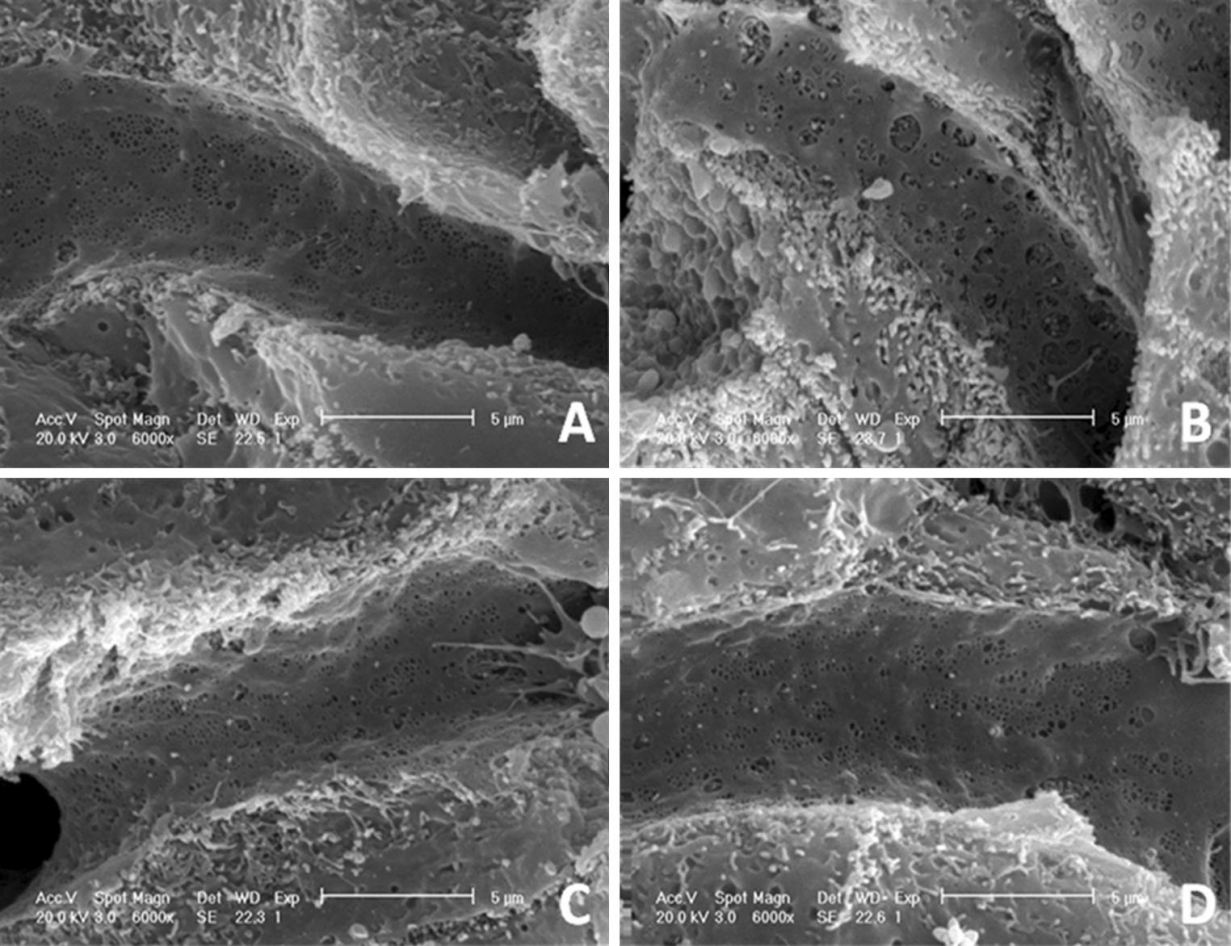
Effects of CMCS and 1,10-phenanthroline on fenestration of LSECs in LPS/D-GalN-induced acute hepatic injury in mice. **a** LSECs in normal mice exhibited fenestration organized in sieve plates with laminar extensions containing abundant rounded pores. Mostly, the pore diameter was approximately 150 nm with a maximum diameter of about 300 nm. **b** Enlarged pores in model control, approximately 500 nm with a maximum diameter of about 1 μm, were obvious larger than that of in the normal group. **c** Smaller pores were in CMCS treatment than that of in the model control. **d** Pore diameter decreased in 1,10-phenanthroline treatment than that of in CMCS treatment. Scanning electron micrographs of LSECs ×6000

**Fig. 6:**
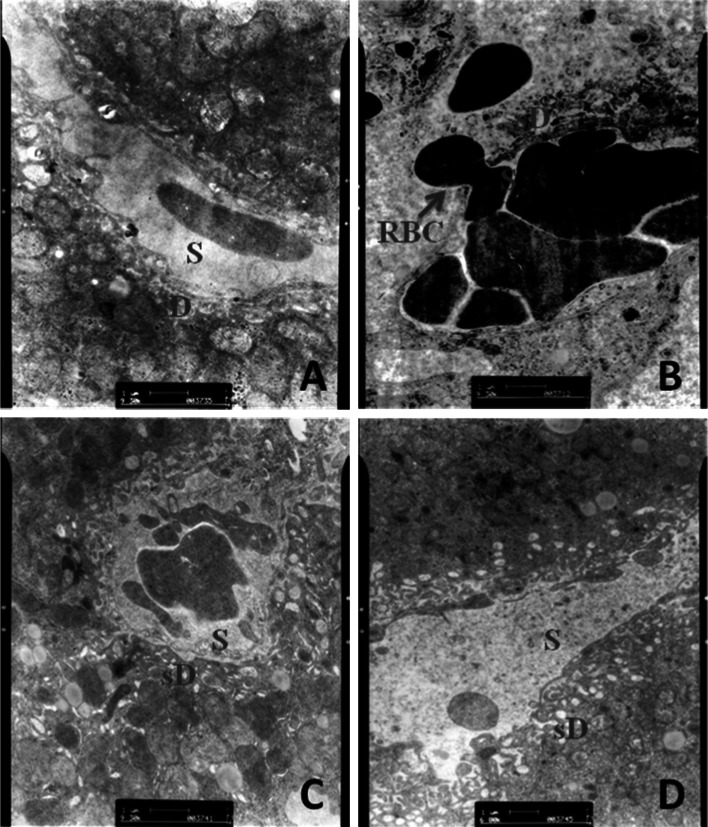
Effect of CMCS and 1,10-phenanthroline on sub-endothelial basement membrane in LPS/D-GalN-induced acute hepatic injury in mice. **a** In normal control, LSECs were thin and flattened cellular expansions. **b** The severe lesioned LSECs in model mice provided with obviously haemorrhagia regions through which RBCs (*red arrow*) from sinusoidal lumen overflowed. **c** LSECS with mildly lesion and small amounts of haemorrhagia were exhibited in CMCS treatment. **d** Relatively unbroken structures were shown in LSECs in 1,10-phenanthroline treatment. Transmission electron micrographs of liver tissue (*S* Sinusoidal lumen, *sD* space of Disse, *RBC* red blood cell). (Color figure online)

**Fig. 7:**
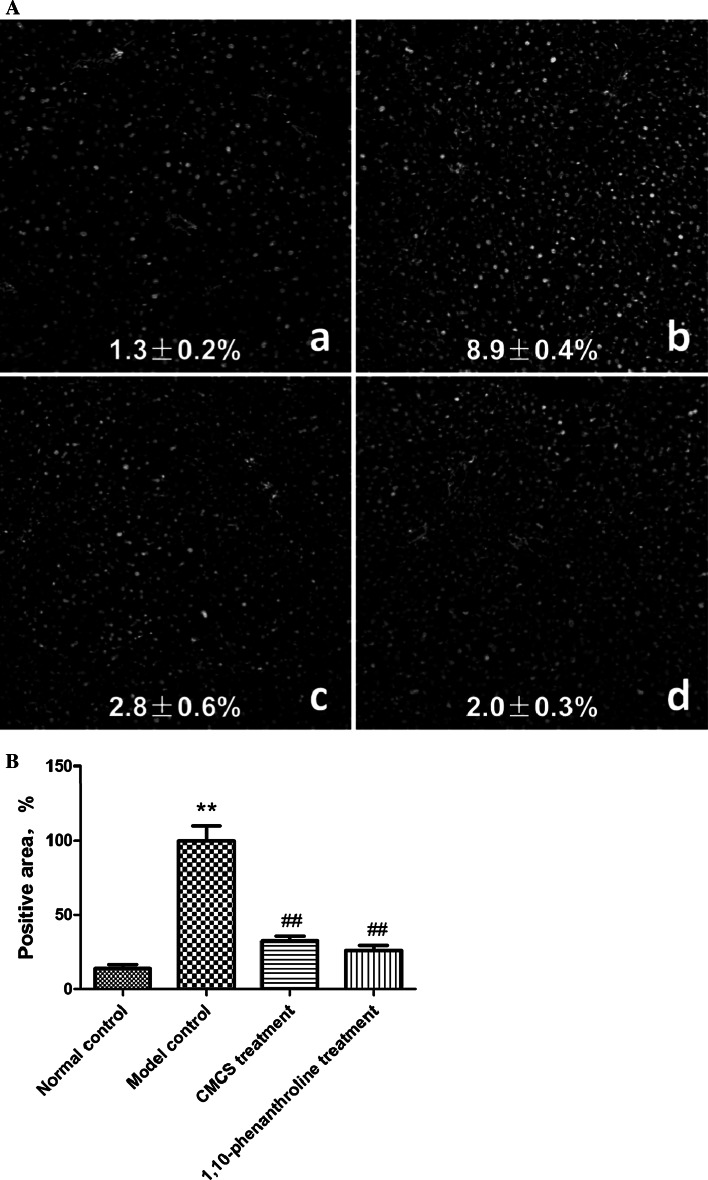
Effects of CMCS and 1,10-phenanthroline on the expression of vWF in LPS/D-GalN-induced acute hepatic injury in mice. **a**: vWF (*red*) around vessels was scarcely observed in normal liver tissue (*a*), while harvest staining of vWF (*red*) was obviously obtained in the model control (*b*). CMCS and 1,10-phenanthroline inhibited vWF expression respectively (*c*, *d*). Immunofluorescence staining ×200. **b** Semi-quantification data for vWF protein level in the liver tissue were calculated in comparison with the model control (100 %) and were shown as percentage of vWF-positive areas; **p < 0.001, compared with normal control; ^##^ *p* < 0.001, compared with model control. (Color figure online)

Herein, we observed the electron micrograph of the LSECs and vWF expression in liver tissue, aiming to survey the potential effect of CMCS on protecting liver sinusoid endothelial cell. SEM and TEM revealed the protective effects of CMCS on LPS/D-GalN induced LSECs injury, respectively. Generally, fenestrations, with laminar extensions containing abundant rounded pores were organized in sieve plates (Fig. 5a) and LSECs were lack of basement membrane, exhibiting very thin and flattened cellular expansions with distally laminal-shaped endochylema (Fig. 6a) in control mice. Besides, levels of vWF in model mice expressed much higher than that of in the normal control (Fig. 7A-a, A-b). After LPS/D-GalN treatment, larger pores of LSECs were exhibited in model mice (Fig. 5b). Meanwhile, red blood cells (RBCs) from sinusoidal lumen overflowed through haemorrhagia regions of severe lesioned LSECs (Fig. 6b). CMCS was demonstrated relatively to reduce the amplification of the pores and to relieve the broken structures of basement membrane, with the mildly lesion and small amounts of haemorrhagia (Fig. 5c). Similarly, 1,10-phenanthroline treatment group showed smaller pores than that of the model control group (Fig. 5d). Furthermore, LSECs from mice treated with 1,10-phenanthroline demonstrated relatively unbroken hepatic scaffold structure (Fig. 6d) Coincidentally, vWF, a golden marker of endothelial function, was virtually increased in CMCS treatment and 1,10-phenanthroline treatment group, respectively(Fig. 7A-c, A-d).

### CMCS prevented LPS/D-GalN decreases in antioxidant defense

In order to evaluate endogenous antioxidant defense, hepatic oxidant/antioxidant system was examined. Levels of tissue ASAFR, ·OH, SOD and GST in mice receiving LPS/D-GalN were significantly higher than those of in the normal control group while tissue MDA levels were significantly lower (Table 2). When compared with the model control group, levels of tissue ASAFR, ·OH, SOD and GST of mice receiving CMCS and 1,10-phenanthroline were lower than those of the model group, respectively. In CMCS and 1,10-phenanthroline treatment group, levels of MDA were significantly decreased, respectively.

**Table 2:** Effects of CMCS and 1,10-phenanthroline on hepatic lipid peroxidation parameters in LPS/D-GalN induced acute hepatic injury mice ($\overline{x} \pm {\text{s}}$)

Group	*n*	ASAFR (U/gprot)	·OH (U/mgprot)	SOD (U/mgprot)	MDA (nmol/mgprot)	GSH (gGSH/l)
Normal control	8	20.98 ± 3.88	487.50 ± 98.07	45.98 ± 5.50	0.94 ± .0.2	187.51 ± 25.38
Model control	12	12.66 ± 2.37**	88.16 ± 24.98**	21.11 ± 5.77**	5.60 ± 0.79**	82.94 ± 14.25**
CMCS treatment	10	16.54 ± 1.41^#,^^	348.07 ± 40.94^##,^^	40.93 ± 4.49^##^	1.72 ± 0.28^##,^^	182.62 ± 13.90^##^
1,10-Phenanthroline treatment	9	19.12 ± 2.26^##^	392.7 ± 49.3^##^	45.30 ± 3.77^##^	1.02 ± 0.24^##^	186.21 ± 20.17^##^

** *p* < 0.001, compared with the normal control; ^#^ *p* < 0.05, ^##^ *p* < 0.001, compared with the model control; ^^^ *p* < 0.05, compared with the 1,10-phenanthroline treatment group

### CMCS downregulated the MMPs activities of liver tissue in acute liver injury

**Fig. 8:**
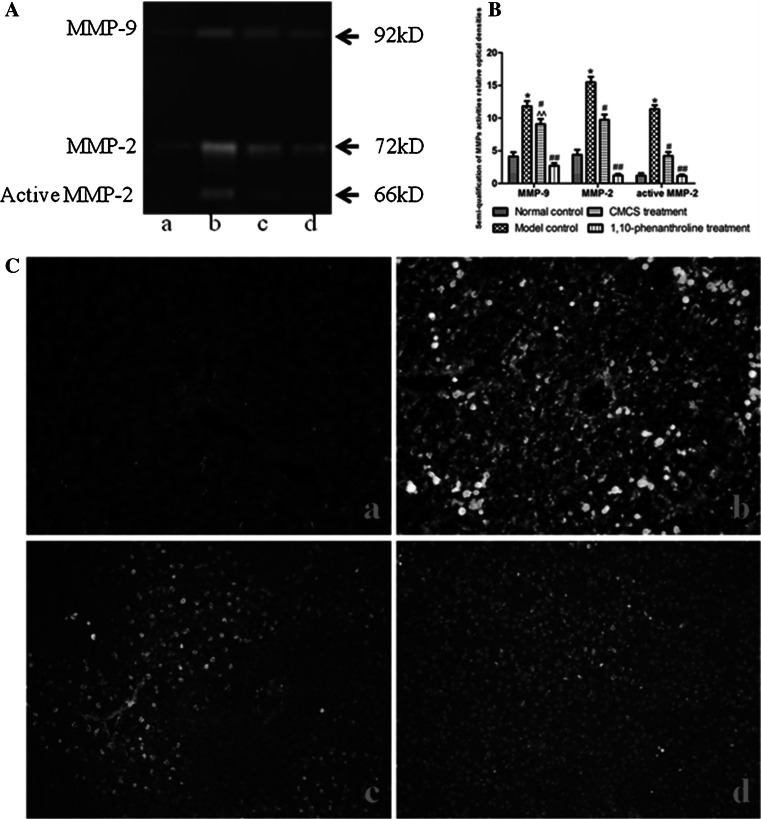
Effects of CMCS and 1,10-phenanthroline on liver MMP-2/9 activities in LPS/D-GalN-induced acute hepatic injury in mice. **a** Assessment of gelatinases (MMP-2 and MMP-9) by gelatin zymography. MMP-9 (92 kDa) and MMP-2 (72 kDa) were respectively present in the first line and second line. The third line showed active MMP-2 (66 kDa). **b** Semi-quantification of MMPs. Data are presented as $\overline{x} \pm {\text{s}}$. *p < 0.05, **p < 0.001, versus the normal control group; ^#^ *p* < 0.05, ^##^ *p* < 0.001, versus the model control group; ^^^^ *p* < 0.001, versus the 1,10-phenanthroline treatment group. **c** MMP-2/9 activities of liver tissue in situ zymography (×200) (*a* normal control, *b* model control, *c* CMCS treatment, *d* 1,10-phenanthroline treatment)

In order to understand the effects of CMCS upon the expression and activity of gelatinases in hepatic tissues, we examined the hepatic MMP-2/9 activities by gelatin zymography (Fig. 8a, b) and in situ fluorescent zymography (Fig. 8c) respectively. The results illustrated significantly lower MMP-2 and MMP-9 activities in the normal control group compared with those of the model control group (Fig. 8b). By contrast, mice treated with CMCS and 1,10-phenanthroline significantly inhibited activities of MMP-2/9 respectively (Fig. 8b). Interestingly, MMP-9 activities and active MMP-2 in CMCS treatment group expressed about two-folder higher than that of in 1,10-phenanthroline treatment group (Fig. 8b).

### CMCS inhibited LPS/D-GalN induced ICAM-1 and VCAM-1 expressions

**Fig. 9:**
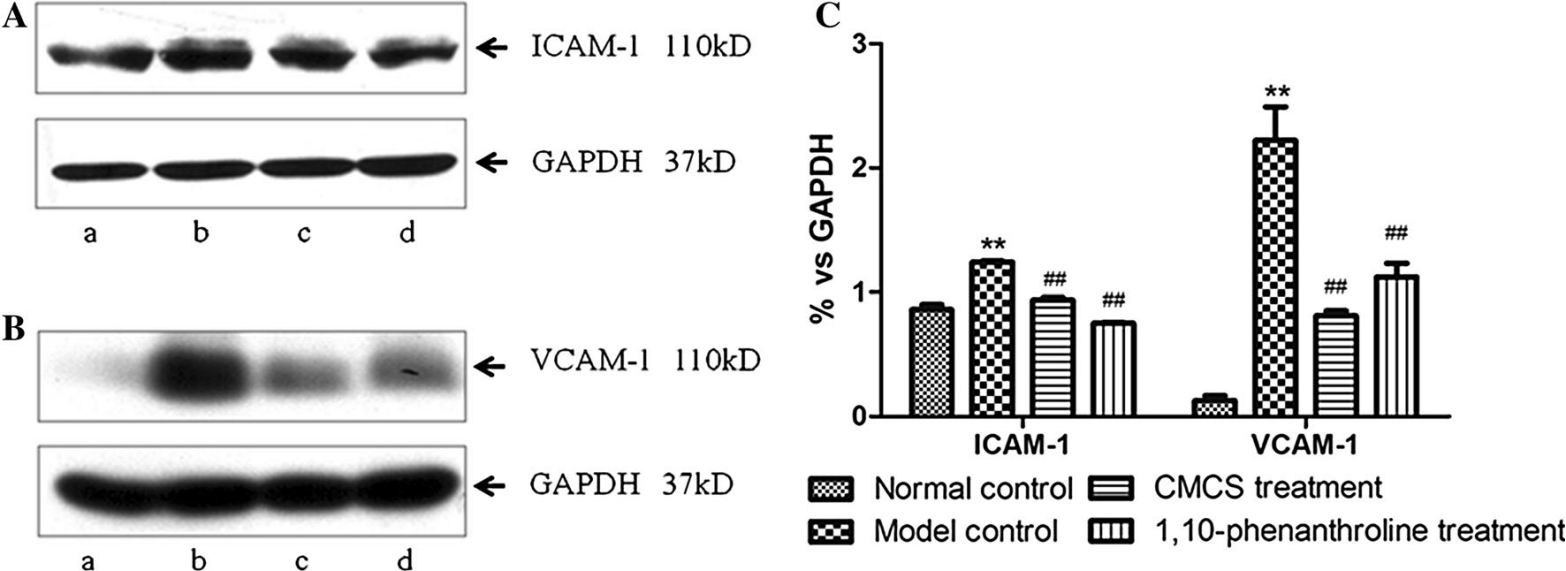
Effects of CMCS and 1,10-phenanthroline on ICAM-1 and VCAM-1 expressions of liver tissue in LPS/D-GalN-induced acute hepatic injury in mice. The expressions of ICAM-1 (**a**) and VCAM-1 (**b**) were determined by western blot (*a* normal control, *b*: model control, *c* CMCS treatment, *d* 1,10-phenanthroline treatment). **c** The *graph* showed semi-quantification of ICAM-1 and VCAM-1 protein expression in related to GAPDH (normal control, *n* = 8; model control, *n* = 12; CMCS treatment, *n* = 10; 1,10-phenanthroline treatment, *n* = 9) **p < 0.01, versus normal group; ^##^ *p* < 0.01, versus model group

Western blot analysis (Fig. 9a, b) showed protein expressions of VCAM-1 and ICAM-1 respectively. Expressions of VCAM-1 and ICAM-1 were significantly increased in model mice while CMCS and 1,10-phenanthroline could decrease the protein expressions of those. Graphic values were represented as the densities of ICAM-1 or VCAM-1 versus GAPDH (%) (Fig. 9c).

## Discussion

Oftentimes, the role of microvascular system in liver injury pathobiology is neglected, although heterogeneous disturbances in portal blood flow have profound consequences on organ function and dysfunctions. Actually, the LSECs, a main cell type of hepatic microvascular system or sinusoids, play a pivot role in maintaining structure and function of the liver. Recent documents demonstrated that LSECs not only were the primary targets of drugs, pharmacological agents and other pathological disturbances, but also might be the initial stimulus for hepatic fibrogenesis [14].

Bacterial LPS, the major component of the outer membrane of Gram-negative bacteria, could induce intrahepatic inflammation to produce various proinflammatory cytokines by activation of Kupffer cells [15]. D-GalN, as a specific hepatotoxic agent, was used to increase the susceptibility to the lethal effects of endotoxin [16]. In the present study, LPS and D-GalN intoxication caused obvious acute liver injury in mice, demonstrated by the elevated serum ALT and AST activities, obvious liver inflammation lesion, widely vWF expressions and scaffolds structure broken among sinusoids, etc. However, CMCS and 1,10-phenanthroline (MMPs inhibitor) could ameliorate the liver injury compared to the model control, evidenced by the decreasing levels of serum liver function, attenuating the inflammation with widely mononuclear cell infiltration, apoptotic bodies, necrotic and cytolytic hepatocytes in liver. Furthermore, CMCS and 1,10-phenanthroline could maintain the hepatic scaffold structure from breaking. Significantly, CMCS treatment could inhibit the expression of vWF with increased level, which might be an original finding in the liver disease and the study of its mechanism. It indicated that CMCS had potential effects against acute liver injury as well as hepatic microvasculature damage. Remarkably, 1,10-phenanthroline acted as a better role in protecting hepatocytes and hepatic scaffold structure than CMCS.

LSECs, as the major cell types of microvasculature, are very sensitive to toxins from circulation. In the normal liver, LSECs were located on the hepatic sinusoid walls, with un-continuous matrix membrane under LSECs (scaffold structure) and fenestrations among cytoplasm. LSECs constitutively expressed the intercellular adhesion molecule-1 (ICAM-1), which along with vascular cell adhesion molecule-1 (VCAM-1), were up-regulated by inflammatory stimuli either in a direct manner or by mediators released from stimulated Kupffer cells. Once acute liver injury occured, ICAM-1 and VCAM-1 were consequently up-regulated on the endothelial surface, mediating the transmigration of leukocytes into the liver parenchyma [17]. More importantly, the fenestrations became large and endothelial matrix was broken, which would lead to dysfunctions of microcirculations. In the study, the model mice had larger pores were presented in LSECs under SEM and damaged endothelial matrix under TEM observation, with a lot of haemorrhagia and broken scaffold structure stained by silver solution under light scope, and increased expression of ICAM-1 and VCAM-1. However, we found that both CMCS and 1,10-phenanthroline could restore the fenestration, decrease haemorrhagia in sinusoids, attenuate scaffold structure damage, and down-regulate the expression of ICAM-1 and VCAM-1. These results indicated that CMCS might have the effects of protecting LSECs from liver injury and keep the structural integration of hepatic sinusoids.

LSECs injury was associated with its detachment from matrix membrane, which was mainly regulated by MMP-2/9. In our study, 1,10-phenanthroline, an MMPs inhibitor, was selected as the positive control. As expected, CMCS could decrease the expressions and activities of MMP-2/9 of model mice, which demonstrated that CMCS down-regulated MMP-2/9 activities, and protected endothelial matrix degradation, resulting in protection of LSECs detachment and injury. Besides, LSECs are very vulnerable to oxidative stress, in the experiment, the hepatic lipid peroxidation in the model mice was obviously demonstrated by decreased SOD and GSH but increased MDA etc., while in CMCS and 1,10-phenanthroline treatment group, levels of MDA were decreased and ASAFR, ·OH, SOD and GST were increased significantly, respectively. All these data revealed that anti-oxidation might be another action mechanism of CMCS against LSECs injury. In the study, we also found that 1,10-phenanthroline could relieve the acute liver injury by down-regulating levels of serum liver functions, protecting hepatocyte from lesion, resisting oxidative stress, etc., suggesting this MMP inhibitor might had potential application in LPS/D-GalN induced acute liver injury.

LPS alone or combined with D-GalN could induce the liver injuries with complicated mechanism. In addition to peroxidative stress and MMP-2/9 regulation, it could mediate the TLR-4 and NF-κB signaling pathway, inhibit the expression of vWF with increased level, and result in regulation of ICAM-1 and VCAME-1 expression and LSEC injury. In the current study, we focused on the effects of CMCS on LSECs in acute injured liver, and the action mechanisms relating to MMP-2/9 activities as well as liver peroxidation. Whether CMCS could regulate LPS induced TLR4/NF-κB signaling pathway remains unknown, this possible underlying molecular action mechanisms of CMCS will be our future investigation.

## Conclusions

CMCS is effective on alleviating LSECs damage and restoring microvasculature integration in acute liver injured mice induced by LPS/D-GalN. Its action mechanism was associated with down-regulation of MMP-2/9 and protection of peroxidation in injured liver.

## Abbreviations

CMCS: Cultured mycelium *Cordyceps sinensis*
LPS: Lipopolysaccharides
D-GalN: d-galactosamine
ALT: Alanine aminotransferase
AST: Aspartate aminotransferase
LSECs: Liver sinusoidal endothelial cells
ICAM-1: Intercellular adhesion molecule-1
VCAM-1: Vascular cell adhesion molecule-1
MMP-2/9: Matrix metalloproteinases-2/9
H&E: Hematoxylin and eosin
TEM: Transmission electron microscope
SEM: Scanning electron microscopy
ASAFR: Antisuperoxideanion
·OH: Hydroxyl free radical
SOD: Superoxide dismutase
MDA: Malondialdehyde
GST: Glutathione *S*-transferase
TUNEL: Terminal deoxynucleotidyl transferase-mediated dUTP nick end-labeling
DAPI: 4′,6-Diamidino-2-phenylindole
vWF: von Willebrand factor
